# Development and Evaluation of a Polymer Composite Material Reinforced by Tectona Grandis Fiber, with Static Analysis

**DOI:** 10.3390/polym17050634

**Published:** 2025-02-27

**Authors:** Sandeep Bavanam Nagaraja Reddy, Kishor Buddha, Kadiyala Chandra Babu Naidu, Dudekula Baba Basha

**Affiliations:** 1 Department of Mechanical Engineering, School of Technology, GITAM University, Bangalore 562163, Karnataka, India; snagaraj2@gitam.edu; 2 Department of Physics, GITAM Deemed to be University, Bangalore 561203, Karnataka, India; chandrababu954@gmail.com; 3 Department of Information Systems, College of Computer and Information Sciences, Majmaah University, Al’Majmaah 11952, Saudi Arabia

**Keywords:** natural fiber, mechanical properties, tensile strength, Tectona Grandis, hardness properties, scanning electron microscopy

## Abstract

This research seeks to investigate the viability of using Tectona grandis wood powder as a reinforcement material in polymer matrix composites because of the increasing awareness of natural fibers that offer impressive characteristics and cost-effectiveness in addition to being biodegradable. The fibers were mixed with epoxy resin, and the mixture was passed through a filter to remove fiber bundles and then compression molded to form composites, which were cured in an oven. Different experiments were performed on the composite to measure its mechanical characteristics. The tests performed were a tensile test to measure the mechanical properties of the material like strength and elastic properties, a compression test for evaluating the behavior of the material under a compressive load, a hardness test for the rate of indentation resistivity, and an impact test for the material’s ability to withstand shock loads. The results showed that fiber reinforcement caused a significant enhancement in the mechanical aspect of the composite, where the compression strength obtained was 249.83 MPa, and the tensile strength obtained was 17.98 MPa. SEM microstructural analysis and a moisture absorption test were performed, while an additional analysis was carried out using Ansys work bench software. This research proves that Tectona grandis wood powder improves the mechanical properties of polymer composites and represents a viable substitute for synthetic reinforcements.

## 1. Introduction

In the past three and a half decades, ceramics and polymers, along with composite materials, have continued to be the most popular materials in the industrialized world. Composite materials applications have increased gradually, penetrating new areas and creating exciting new applications in various industries. Lightweight material needs have been fulfilled by green composites, and thus, more work is currently being done to make them cost-effective. Structural manufacturing and assembly of thermoplastic composites have witnessed considerable innovation in meeting the costs of optimum manufacturing processes. The uses of composites are not limited to aeronautics and the automobile industries but seem to be finding more opportunities, especially in the transportation business, which has proved to have greater space for the use of composites.

The employment of composite materials comprising natural fibers has been expanding rapidly and has a high turnover involving intensive activities and interest both industrially and academically [1]. This is due to the fact that the composites in question are recyclable and environmentally friendly and have the potential to act as substitutes for traditional composites in different fields. From a general point of view of mechanical recycling, fiber-reinforced polymers are shredded, crushed, or milled, and the resulting materials are then classified into powdered products and fibrous components [2]. The recovered materials can be used as filler or reinforcement through reimpregnation with a new resin, or they can be added to asphalts and cement. The resulting particulate grades are mainly utilized as filler, while the fibrous components with higher aspect ratios are utilized as reinforcement. These materials are environmentally friendly and cost-effective and will not be depleted in the near future. Flax, jute, hemp, and bamboo natural fibers, as well as wood, having lignocellulose content, have been known for ages, and are currently being widely used for reinforcement in green composites. Due to the versatility of the raw materials, as well as their low weight, low cost, and useful mechanical characteristics, they can be considered a potential substitute for glass fibers in certain applications. While natural fibers have distinct properties, such as vibration-damping capabilities, they can serve as alternatives to carbon fibers in applications where these characteristics are advantageous. Natural fiber composites are found to be used in transportation systems like railways, automobiles, aviation, construction and packaging, and civil engineering, to mention but a few; they are also used by the military, with environmental concerns topping the list.

The suitability of bamboo as a structural material was assessed by Yuan Feng et al. by studying some static mechanical properties of bamboo [3]. During the tensile testing of male bamboo, they investigated the use of several grip supports in tension tests. The tensile strength of the material was found to have an average value of 96.62 MPa, while Young’s modulus, calculated from the stress–strain curve, was determined to be 20,050 Mpa [4]. These values highlight the material’s capacity to withstand high levels of stress before failure. Bamboo’s high strength-to-weight ratio suggests it could be a useful material in structural applications, including RCC members, provided treatments are applied to mitigate water absorption and improve bonding. Special tests and, even more so, durability tests must be conducted before bamboo is used in sensitive structures. S. Vigneshwaran further notes that present-day composites are more durable than those of previous generations and are used widely in various industries [5]. They present advantages such as high strength-to-weight ratios, toughness, and greater durability. These earlier studies applied natural fibers in composites due to their environmental benefits and because the raw material is cheap. The composites that were produced are made with wood dust as fiber reinforcements and cashew nutshell liquid (CNSL) as the matrix, and the tensile strength of the samples was established.

Research performed by Masturi et al. was centered on producing a composite material derived from Tectona Grandis leaf waste, polyvinyl acetate (PVAc), and fiberglass [6]. The researchers adjusted the material composition to determine the effects of fiberglass on the compressive strength of the composite. From their observations, they realized that the incorporation of fiberglass greatly improved the compressive strength: a 4.8% fiberglass fraction offered suppleness that was 51% higher than that of the inherent composite. This gives an indication of the improvement realized by the use of fiberglass reinforcement in enhancing the mechanical properties of these composites. Natural fibers have been used in India for more than 3000 years, but the use of plastics has increased day by day, and it is a substantial threat to our environment, as addressed by Mamidi et al. [7]. The key idea is to further minimize the utilization of plastics, and one of the available strategies is the creation of new fibers for different industries. Natural fibers are environmentally renewable and affordable relative to synthetic fibers, thus making them ideal. The subject of this research is the concept of an innovative fiber–matrix composite material that can be used to make interior automobile parts. This investigation is centered on employing Tinospora cordifolia and Tectona grandis, both natural fibers that are easily available in India. These fibers have low density, high mechanical strength, and cost advantage over many other natural fibers. Although Tinospora cordifolia and Tectona grandis have medicinal values, no studies have examined their usability in producing composite materials. Secondary resources like sawdust, wood particles, chips, etc., which are by-products of the woodworking industry, can be efficiently used to manufacture composites. Chip block pallets (CBPs) are available as industrial chip blocks and are popular, particularly in the logistics industry, as studied by Dede Hermawan et al. [8]. Specifically, the work investigated the production of CBPs with teak wood biomass using polyurethane adhesive with different proportions and particle size distribution. The production process was divided into two stages: the first stage determined the effect of polyurethane adhesive content on production, and the second stage analyzed particle size composition. Albumin hydrogels with dimensions of 9 × 9 × 9 cm^3^ were produced by the cold-press method, with a density of 0.6 g/cm^3^. The physical and mechanical properties of the CBPs were determined according to NWPCA standards. More specific characteristics involving density/porosity, moisture uptake, dimensional stability, water absorption, CS, and SHS were carefully tested. The analysis of the results showed that specimens with a CBP particle size of 4–14 and polyurethane adhesive content of 4.5% yielded the highest performance, at 14.67 MPa and 371.50 N, in terms of the compressive strength and screw-holding strength, respectively. This is an indication that both the physical and mechanical properties of chip blocks produced from teak wood waste could offer good bearing support for pallet pads, thus yielding greater capacity.

Altlomate et al. investigated the fibrous nature and mechanical properties of Hibiscus sabdariffa fiber for reinforcement in a matrix of urea–formaldehyde (UF) resin [9]. For various fiber volume percentages, they measured the tensile strength, compression strength, and wear-enduring proprieties of randomly arranged Hibiscus sabdariffa fiber polymer composites. The results highlighted a great improvement in the mechanical properties, as depicted by the urea–formaldehyde resin–Hibiscus fiber composite. A study was also conducted on the density and thermal analysis of the resin and the biocomposites. The tensile properties of bamboo lamina were analyzed from the data obtained from the tensile tests performed Abdul Khalil et al. with bamboo samples cut from various regions of the bamboo culm, starting from the middle to the outside section [10]. Rule of mixtures analysis and linear regression analysis were used to analyze the results, which showed that the strength of the fiber increased from the base to the top, and conversely, the strength of the matrix decreased from the base to the top. To enhance the use of bamboo in practical projects, the bamboo lamina was transformed into layered laminate bamboo composites (LLBCs) through adhesive bonding. The ASTM D3039 [11] standard was used to determine the static mechanical characteristics of the LLBCs with reference to those of wood fibers and the tensile failure mechanism of both the laminas and the LLBCs, as examined via SEM. Sisal fibers were also investigated by D. Getu et al., and the highest impact strength was reported compared with lignocellulose fibers and average flexural as well as tensile strength [12]. The study also contained a systematic evaluation of the effects associated with sisal composites, especially their physical and mechanical properties. The research data showed that these composites, particularly the ones reinforced with sisal fibers, possess a wide possibility of being used in the automobile and transport sectors. This is mainly attributed to the low density and high specific strength of the sisal fibers necessary to produce lightweight but strong materials.

The composite structures found in fiber-reinforced polymers exhibit a synergistic complexity that stems from their numerous advantages, especially in low-weight applications. In their synthesized analysis, Thandavamoorthy et al. incorporated and quantified some vital parameters, including surface morphology, mechanical properties, and water absorption, of bran particle composites reinforced with carbon, basalt, and Kevlar fibers using a hand lay-up technique [13]. to the study compared process parameters and field measurements and provided essential characteristics such as water absorption, impact energy, tensile strength, and flexural strength. When the researchers redesigned the stacking sequence by adding three types of fibers for laminate and increasing the percentages of Kevlar and carbon, enhanced performance characteristics were achieved and attained an impact energy of 6 Joules, a tensile strength of 183.10 MPa, and a flexural strength of 352 MPa. These enhanced mechanical properties position the composite material for use in demanding applications like aerospace components and bullet-proofing.

The current work presents experimental and composite processing techniques aimed at characterizing and assessing mechanical properties. It highlights the macro and micro features of a composite material made from fibers extracted from Tectona grandis wood powder. This article discusses the methodologies for fabricating these composites and the experimental approaches utilized. The experimental design and testing methodology employed in this study were sufficient to provide a comprehensive evaluation of the mechanical properties of the composite material, including tensile strength, compression strength, hardness, and impact resistance [14]. Apart from the energy dispersive spectroscopy, the present work provides scanning electron microscope (SEM) data and moisture absorption data regarding the composite. This has given a high tendency to employ natural fibers rather than synthetic fibers when applying fiber-reinforced composites for sustainable engineering systems. This is due to its ecological advantages such as biodegradation and recyclability associated with natural fiber composites, which are added features of high stiffness and specific strength. However, these composites face certain challenges, including moisture susceptibility, inadequate fire ratings, limited hardness, and intricate processing methods. This study particularly focuses on the issue of weight variation when producing fibers from Tectona grandis wood powder. Previous literature indicated weight fractions between 30% and 50%, revealing that higher fiber weight can result in increased delamination [15]. Accordingly, the present study builds upon the preliminary results published in [15], including additional data and a more comprehensive analysis, where weight fractions of 35%, 45%, 55%, 65%, and 75% were utilized for the composites. Complementing the experimental work, static analysis was conducted using ANSYS Workbench 2024R2 (student version), which allows simulation and predicts how the composite will respond to static loads.

## 2. Materials and Methods

The concept of particulate composites revolves around reinforcement using particle-shaped elements rather than traditional fibers. These particles can exhibit shapes such as cubes, spheres, or irregular geometries, and while they may not provide substantial improvements in fracture toughness, they play a key role in increasing the composite’s stiffness. Particulate fillers are frequently employed to upgrade the matrix material, offering numerous advantages, such as enhanced electrical and thermal conductivity. These composites are also designed to withstand extreme temperatures, improve machinability, and increase surface hardness and wear resistance. By minimizing shrinkage, they maintain dimensional stability, making them better applications in industries that demand durability and precise performance, like electronics and automotive manufacturing.

**Figure 1:**
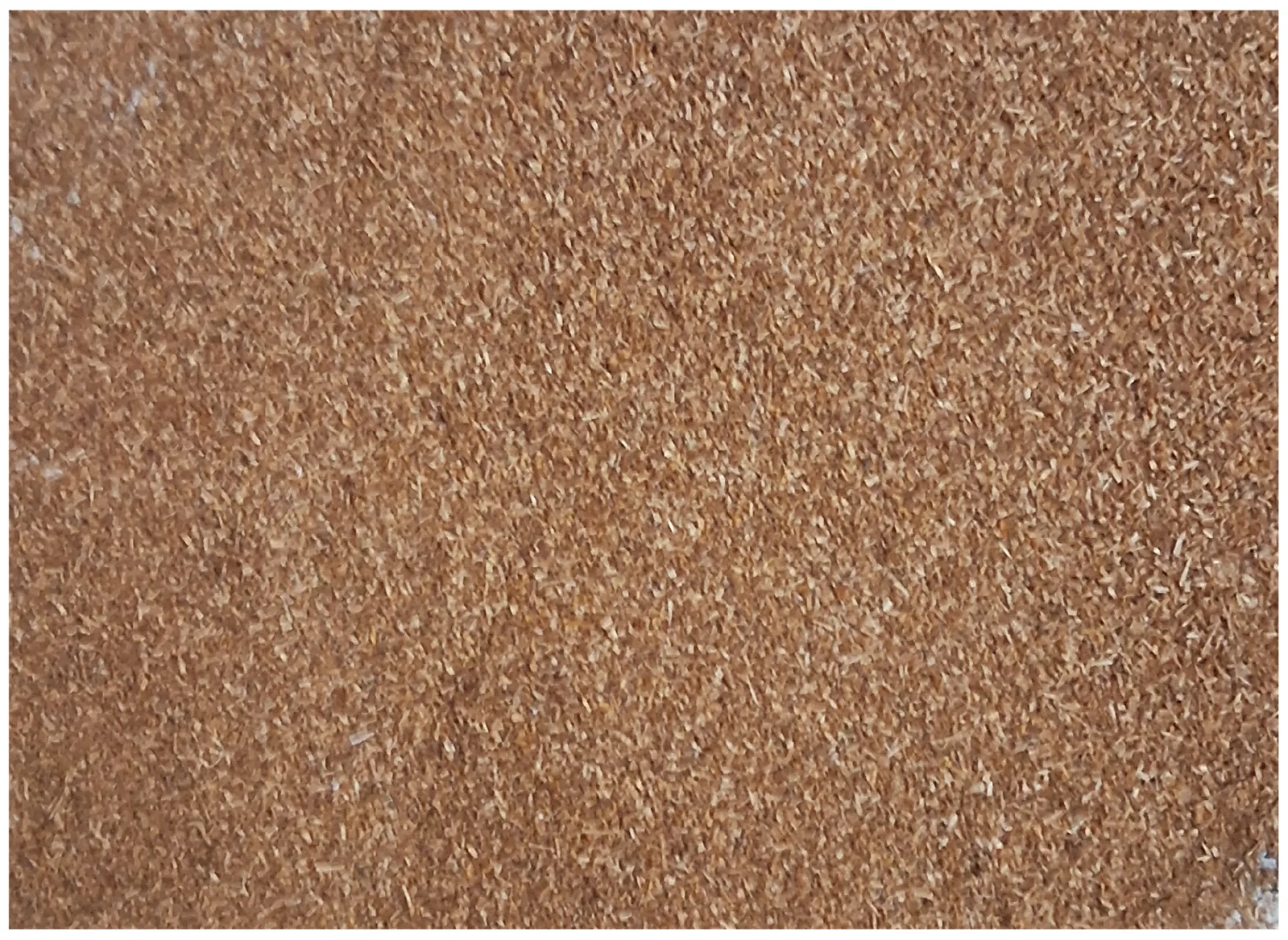
Wood powder of Tectona grandis.

The current study uses Tectona grandis wood powder fibers as reinforcement, as depicted in Figure 1. The process includes a comprehensive explanation of the preparation and experimental setup needed for green composite fabrication and mechanical testing. The Tectona grandis wood powder was purchased from a local timber merchant, and the sample was placed in a closed vacuum chamber and dried for two hours at fifty degrees Celsius. The particle size of the wood powder was controlled during processing, with a maximum limit set at 500 μm, ensuring consistency throughout the composite material. This method ensures that the composite is ready for mechanical performance evaluations.

### 2.1. Preparation of Specimen

Composite material fabrication was achieved using the compression molding method. The epoxy resin used was Araldite LY556 (HUNTSMAN ARALDITE, Meerut, Uttar Pradesh, India), combined with the hardener HY 951 at a 10:1 weight ratio, allowing the mixture to cure at low temperatures. In the first composite formulation, the weight fraction was composed of 35% Tectona grandis wood powder and 65% epoxy resin. Some of the materials used in the composites had to be manually mixed to realize proper dispersion of fibers into the matrix. For the next set of composites, the weight fraction was increased to 45%, and the resin was reduced to 55%. Similarly, the weight fraction of Tectona grandis wood powder was increased further to a 75% weight ratio. The composite panel is presented in Table 1 according to the specifications and weight fractions.

**Figure 2:**
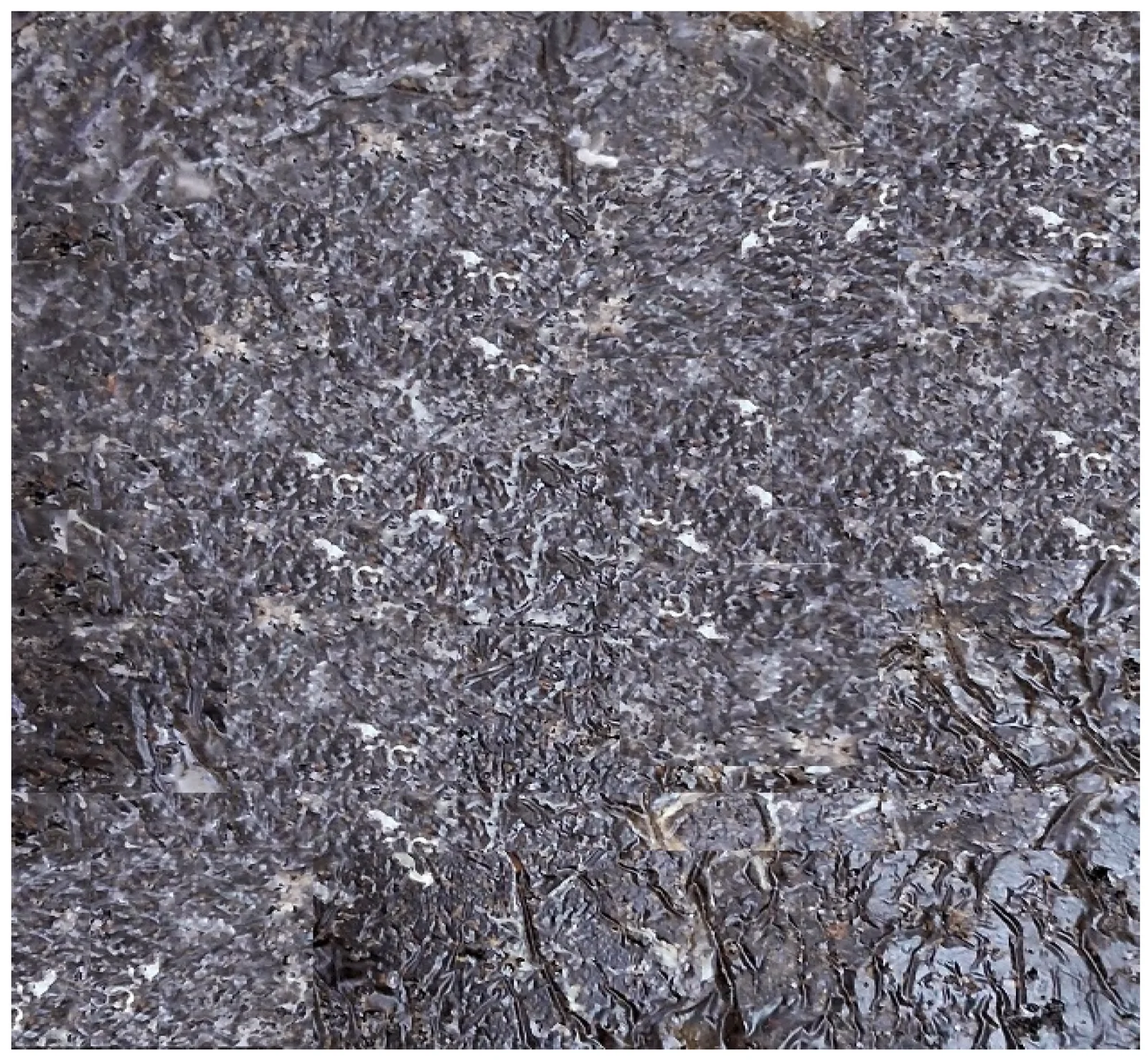
Tectona grandis wood powder fiber is used in the fabrication of composite panels.

Once prepared, each composite was subjected to a 50 kg load and cured for 24 h before being removed from the mold. Both a first curing at 80 °C and a further post-curing under vacuum at 120 °C were introduced to provide the polymer matrix with better mechanical characteristics and thermal resistance. The first curing stage provided a chance for inter- and intra-chain crosslinking of the polymer chains; the second stage of curing under the help of vacuum facilitated additional polymerization of the organic residues, if any. This approach was used based on experimental analysis done during the material development process, where it was discovered that a two-step curing cycle is beneficial in increasing the structural formations’ mechanical strength and the durability of the material. The demolded composite was further processed in a vacuum oven for further post-curing, which took 2 more hours. Rectangular composite panels were produced, and samples were carefully cut using a diamond cutter to obtain smooth surfaces for subsequent mechanical testing [16]. Figure 2 shows the finished composite panel made with Tectona grandis wood powder fibers (The authors convey thanks to QuillBot’s free online AI grammar checker for correcting English of the Materials and Methods section).

### 2.2. Tensile Strength Test

This test is designed to measure the force that can be resisted by a material when an applied load is perpendicular to its axial length, assessing the material’s capacity to stretch before it ultimately breaks. This test affords a basic notion of the technical characteristics of the given material and its performance when under tensile stress. ASTM standard D638: type III requires the use of a specimen in the form of a dumbbell to be used in assessing reinforced composites [17]. The tests were conducted with a Computerized Universal Testing Machine (UTM) (model Zwick/Roel, Worcester, UK) to obtain accurate testing of mechanical properties [18]. With this advanced equipment, it was possible to determine, with reasonable precision, parameters such as modulus of elasticity, tensile strength, and other key features of the composite material [19].

**Figure 3:**
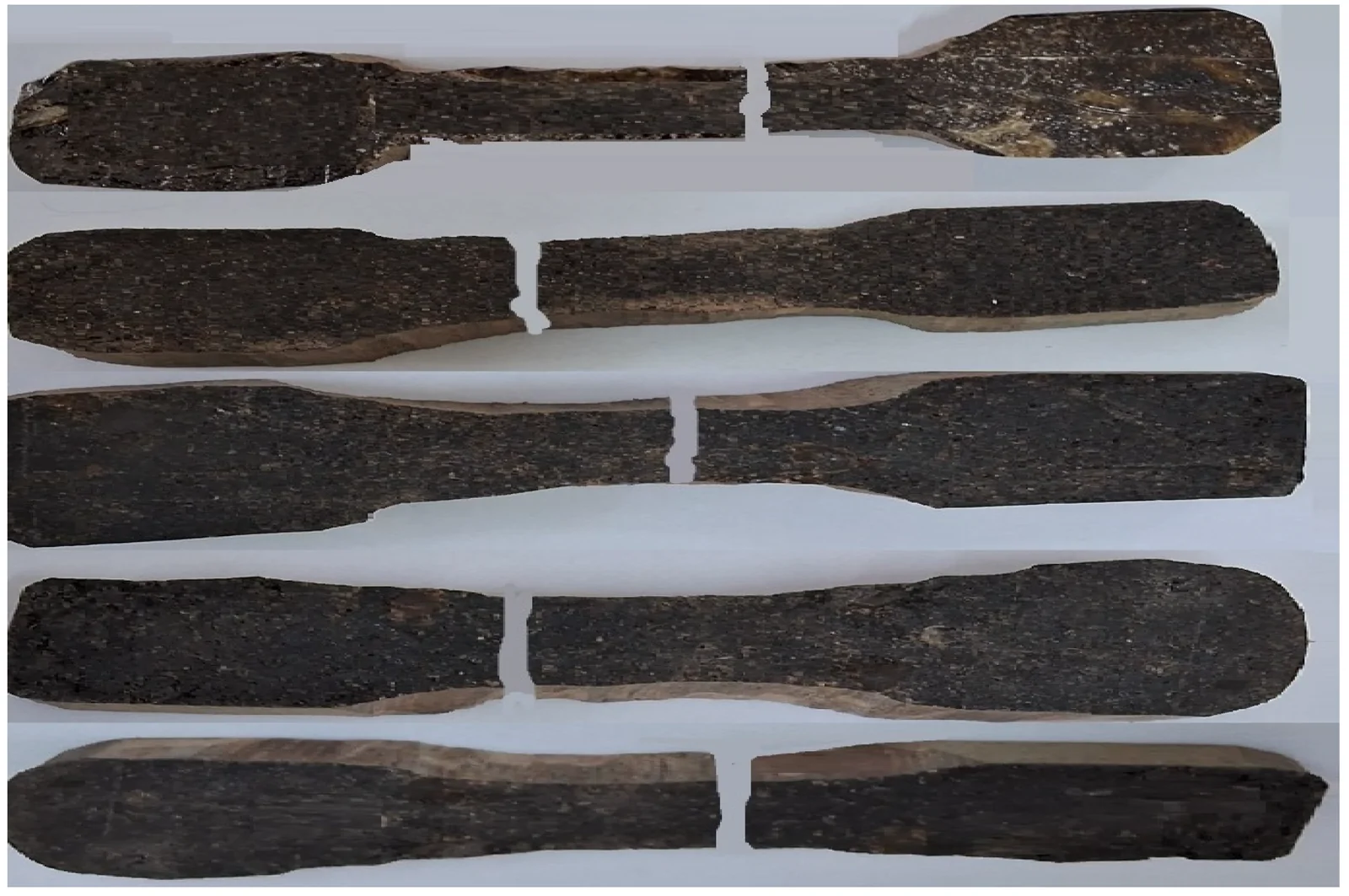
Tensile test specimens of Tectona grandis wood powder. Fiber composites with different weight fractions (%).

In the test, a long specimen of 165 mm was placed vertically between the lower and upper jaw of the machine. Then, 25 mm of the jaws gripped each end of the specimen while the tensile force was applied to the middle gauge length. Before the test, the extension of the extensometer/expander was brought to zero, and the test was performed at a constant straining rate of 10 mm/min [20]. This test was carried out while pulling at the two extremities in a longitudinal direction until the specimen fractured. In the test, the force applied was used to obtain the new dimensions, especially the extension of the gauge section. It was possible, after the test, to note the ultimate tensile load and the deformation at that load. The test panels were prepared to the necessary dimensions specified by ASTM standard D638 for the tensile test of composite materials. Specimens for tensile tests prepared from the composite with varying weight fractions are shown in Figure 3.

### 2.3. Compressive Strength Test

The static load capacity of the composite was assessed using a Computerized UTM, specifically the Zwick/Z150 model. This advanced equipment was employed to evaluate the compression strength of the specimens, as indicated in ASTM D695 [21,22]. In this evaluation, short composite specimens, usually with a square-shaped cross-sectional profile, were used. The specimen was carefully oriented and placed in the central test region of the UTM so that the load could be applied appropriately. In the testing procedure, the crosshead was moving at a speed of 10 mm/min, and a uniaxial compressive force was applied to the specimen until failure was achieved. The compression testing machine automatically recorded force-displacement data throughout the entire testing process, providing detailed information on the mechanical behavior of the material. To determine the composite’s compressive strength at its failure point, the maximum load applied just before rupture was split up by the initial cross-sectional area of the sample [23]. This calculation provided a precise measurement of the material’s ability to resist compressive forces. In addition, the extent of deformation observed in the sample during the test was used to calculate the percentage of strain endured, offering insights into the material’s ductility and deformation behavior under stress. Based on such analysis, it is possible to gain important information on the failure mechanisms and mechanical response of composite material under a compression loading state.

### 2.4. Hardness Test

The Rockwell hardness test was utilized in this study to evaluate how well the composite surfaces could withstand penetration when subjected to an applied static load. The test employed a direct reading instrument that adopted the differential depth measurement approach. Firstly, a small load was added to set up a reference level. This was followed by a peak load for a specified time, and then the minor load is the only remaining load. After imposing a major load and calculating the depth variation, the Rockwell hardness number was established [24]. This measurement gives a quantitative value for the hardness of the material under test, which in its basic sense is the ability of the material to resist indentation under a specific force. Indeed, the entire process takes approximately 4–5 s. Due to its suitability in testing soft materials such as fully annealed steels, composites, and non-ferrous materials, a Rockwell hardness tester with an indentation ball of ¼ inch diameter of a hardened steel ball was used in the present study. For this research, the cyclic load control test was performed following the ASTM E18-20 standard procedure [25]. To assess the behavior of the material under cyclic loading conditions, a minor load of 10 kg was applied, which was succeeded by a major load of 60 kg [26]. This created standards to make sure that proper means were followed for measuring hardness and durability efficiently.

### 2.5. Impact Test

Based on ASTM standard D296, the Charpy test is considered the basic method for evaluating the capacity of a composite material to withstand shock loads or impact [27]. This test reveals how much energy the material is capable of attaining at the moment of specimen failure. This process entails the utilization of a drop weight or pendulum in order to strike the surface of the composite material and measure the energy of the event [28]. The results yielded during this testing approach give a measure of the shock resistance of the composite material that is capable of sustaining impacts of high energy without or with minimal cracking or deformation. Based on these properties, the test enables identification of the general stiffness and ability of the composite to withstand actual use conditions that it will be subjected to. The test procedure to be followed is based on generating a Tectona grandis natural fiber composite and subsequently applying an impact load on the fiber mounted with a descending pendulum or falling weight tester from a standard height. The outcome gained from this test is a measure of the ability of the material to absorb energy and a total measure of its impact strength. This test is common in the auto industry, sporting goods industry, and protective clothing industry, where impact strength is normally high.

### 2.6. Moisture Absorption Test

This test was performed following the guidelines outlined in ASTM D570, which specifies the procedure for immersing specimens in water for a defined duration of time to evaluate moisture absorption characteristics [29]. This procedure forms the basis of the research work presented herein, where the effects of water absorption on natural fiber composite materials were investigated. This general form of testing makes it possible to assess the moisture absorption behavior of these composites, which is essential when estimating the durability and reliability of these composites under various environmental conditions [14]. First, the composite samples were left with the lid closed for one hour at 50 °C in a microwave oven with a view to drying the samples. They were then allowed to cool to a moderate temperature and then weighed so as to obtain their mass at time zero. Solvent swelling involves putting these dried samples in distilled water at a range of temperatures for certain durations of time. The samples are removed after 24 h, and surface water was mopped with a piece of cloth or tissue paper. The specimens were weighed at 24, 48, 72, 96, and 120 h intervals on a Graduate digital scale with divisions of 0.01 mg [30]. This process continued until the samples reach their saturation point. The weight gain was calculated by comparing the sample’s initial and final weights. Finally, the % of moisture content was visualized against the time of immersion to analyze the bi-surface composites’ moisture absorption profile.

### 2.7. Scanning Electron Microscopy (SEM)

SEM is one of the most effective methods for morphological fiber analysis, as it is the most popular technique in surface analysis [31]. High-resolution SEM has proven invaluable in studying surface topology and analyzing failures. This method enables visualization of fiber orientation in thermoplastic-reinforced composites and offers insight into fiber-matrix bonding [32]. SEM gives a qualitative 3D visual representation of surface characterization and quantification of surface roughness. In this technique, a fine probe of electrons is directed at the object’s surface, and secondary electrons are emitted, creating an intensity profile proportional to the surface profile. While SEM works for all materials, non-conductive surfaces should be coated with a mild layer of conductive materials, such as platinum or gold, to ensure accurate results. The resolution of topographic details can reach up to 3 nm. SEM remains a powerful tool for surface analysis before more advanced methods are employed [33].

### 2.8. Static Analysis in ANSYS

ANSYS is a modern and complete finite element program that has capabilities in analyzing various physics fields, for example, static structural, nonlinear, buckling, vibrational, fluid flow, thermal, implicit and explicit dynamics, hydrodynamic flow, electric field, and electromagnetic analyses. It can also conduct coupled field analysis by consolidating one or more different physical analyses. ANSYS is an incorporated system, with all operations carried out under one GUI. Establishing the model, running it, and post-processing the results are all managed without leaving the ANSYS environment.

The layered composites of engineering involve several definitions that involve different materials, thicknesses, and measurements. Of course, the engineering question here is: how efficient will this be when implemented or when in the working environment? It also includes tensions and distortion, several failure theories, and various failure theories. Owing to the fact that Composite Prep-Post is a preparation and post-processing software program included in ANSYS, it has every single necessity one needs for the analysis of composite structures and materials. ACP is an add-on to ANSYS Workbench and integrates directly into the design environment as a set of composite tools attached to the standard analysis tools. As such, the whole workflow for the composite structure can be achieved from design to exhibiting final information production, as described above. The geometry of tooling surfaces of a composite structure is also employed for analysis and manufacturing. According to this geometry, FE meshing and boundary conditions, as well as composite description, are implemented on the structure at the pre-processing level. Post-processing is used after a completed solution, with the objective of evaluating the performance of the design and the type of the material. If the design is insufficient, or the material is not adequate, the geometry or material has to be changed, and the highest frequency is performed again.

## 3. Results and Discussion

This research study focuses specifically on examining the mechanical properties of wood fiber-reinforced composite materials. By investigating the surface characteristics, the study aims to understand the fiber–matrix interaction, the surface roughness, and the influence these factors have on the composite’s overall performance. This work encompasses the detailed process of producing these composites and conducting a series of experimental tests. Tensile, compressive, and hardness test studies were carried out; impact strength was assessed, as well as testing of the material’s moisture absorption capability. Further, this study established the surface topological features of the composites using SEM. This research also entails the performance of extensive computational analysis using ANSYS Workbench to predict and study the mechanical behavior of composite specimens in different situations.

### 3.1. Effect of Composite Fiber Reinforcement on Tensile Strength

**Figure 4:**
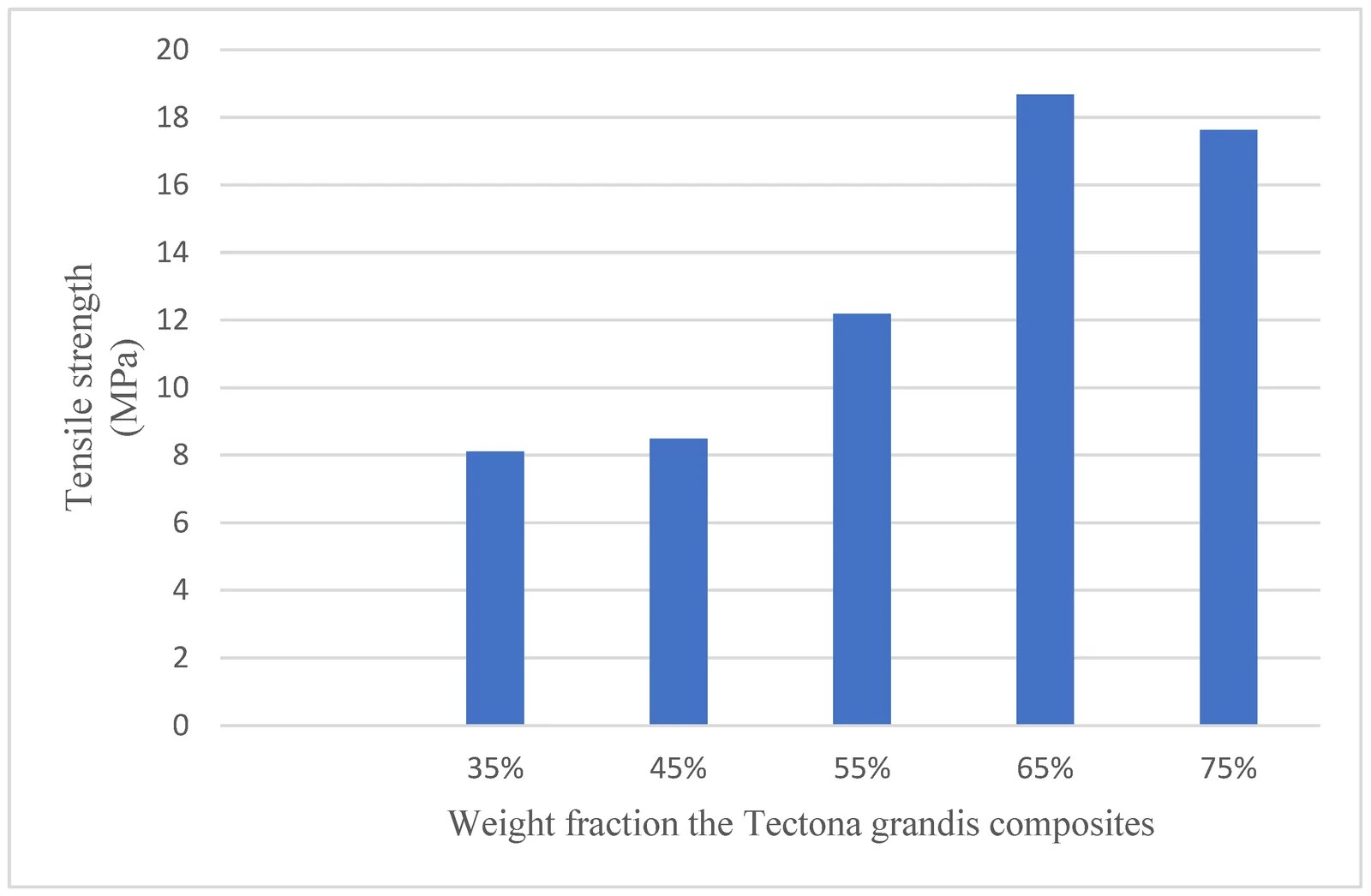
The impact of tensile stress on the weight fraction of Tectona grandis wood powder fiber composites.

Figure 4 gives a comparison of the tensile strength of Tectona grandis wood fiber composite at various weight fractions. The recorded values of the tensile strength of these composites varied indiscriminately, in the range of 8.11 MPa to 18.68 MPa for stress load. Furthermore, the composites exhibited a maximum tensile stress of 18.68 MPa, which reflects their structural integrity and ability to bear applied tension load under the given test conditions. These outcomes show how the weight fraction of fiber impacts the mechanical characteristics of the composite, showing the relationship between composition and load capacity. The results obtained from the experiment show that tensile strength increases with fiber weight fraction, and that the fibers are critical for strengthening the composite. The decline in tensile strength at the highest fiber loading (75%) can be attributed to fibers tending to agglomerate due to insufficient dispersion, leading to stress concentration points. Higher fiber content may reduce the availability of the resin matrix to fully wet the fibers, resulting in weak fiber–matrix bonding. Poor wetting was observed as the filler weight fraction increases beyond 65%. By this reinforcement effect, the mechanical performance of the composite material was enhanced, in that tensile forces are easily transferred between the fibers and the matrix. This interaction equitably shares stress between the fibers and the matrix, which, in turn, increased the composite’s carrying capacity [34]. The optimized stress transfer mechanism enhanced the tensile strength of the material under static loading conditions. However, the reduction in tensile strength and strain at break observed at the highest filler weight fraction suggests a potential decline in stress transfer efficiency, possibly caused by filler agglomeration and limitations in interfacial bonding. Table 2 presents the tensile mechanical properties and tensile loading behavior by tensile testing of the material.

### 3.2. Effect of Composite Fiber Reinforcement on Compression Strength

**Figure 5:**
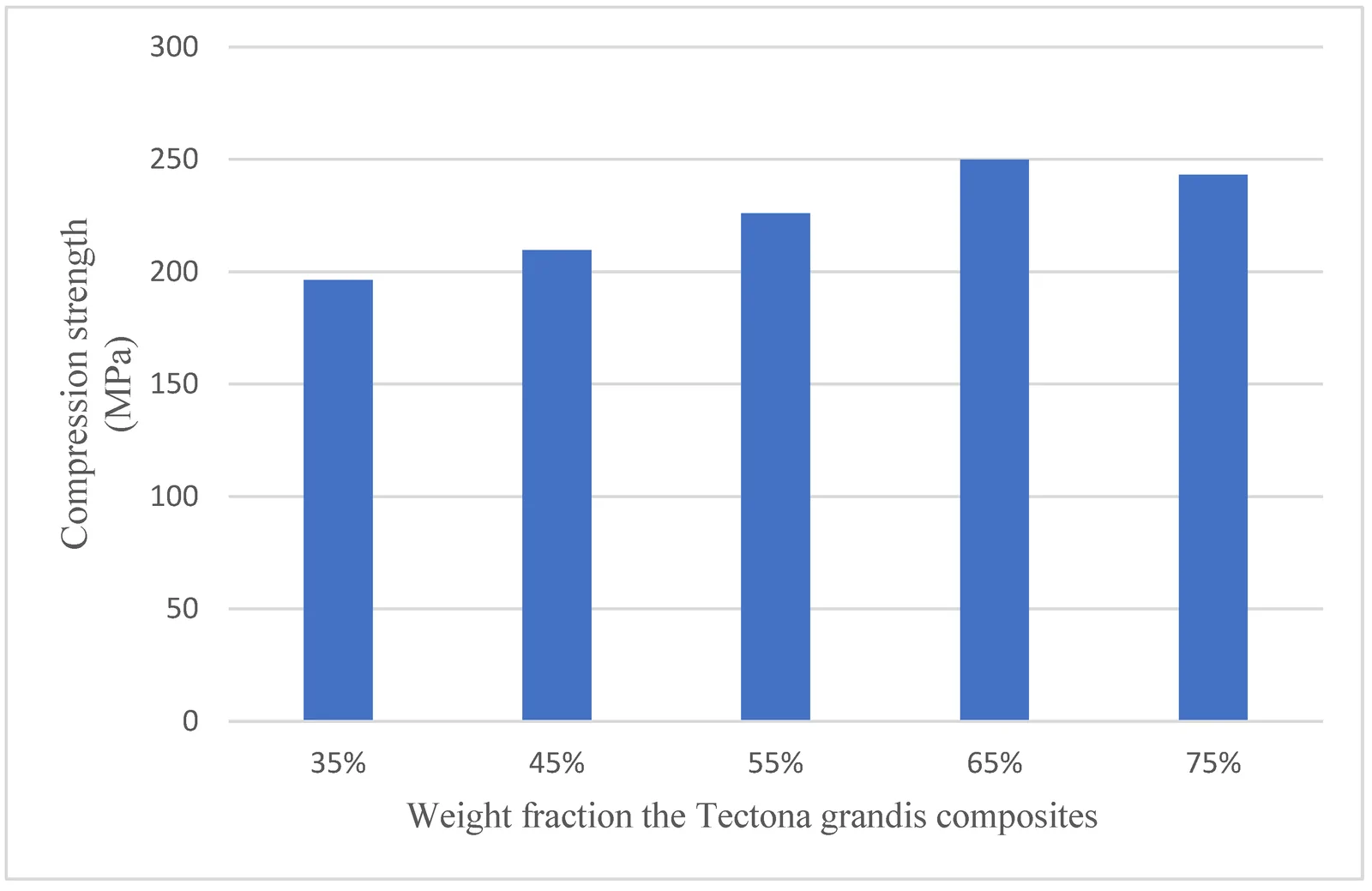
Influence of compression stress on the weight fraction of Tectona grandis wood powder fiber composites.

The compression strength of the Tectona Grandis wooden fiber composites with respect to various fiber weight fractions is given in Figure 5. This figure illustrates how various weight fractions affect the load-carrying capacity of the composite in terms of compression loads. Studying the dependence of the mechanical characteristics of the composite on the content of fiber inclusions provides significant information for developing further strategies to improve load compression [35]. The compression strength results for the composite materials show the values were between 196.32 MPa and 249.83 MPa, with a maximum load-carrying capacity of 24,900 N. According to the test results, there was a steady rise in compression strength as the fiber weight fraction rose, meaning that the fibers act as reinforcement for the composite structure. This improved the material’s capacity to bear compressive loads. The specific results for some weight fractions are presented in Table 3 below.

### 3.3. Effect of Composite Fiber Reinforcement on Hardness Test

**Figure 6:**
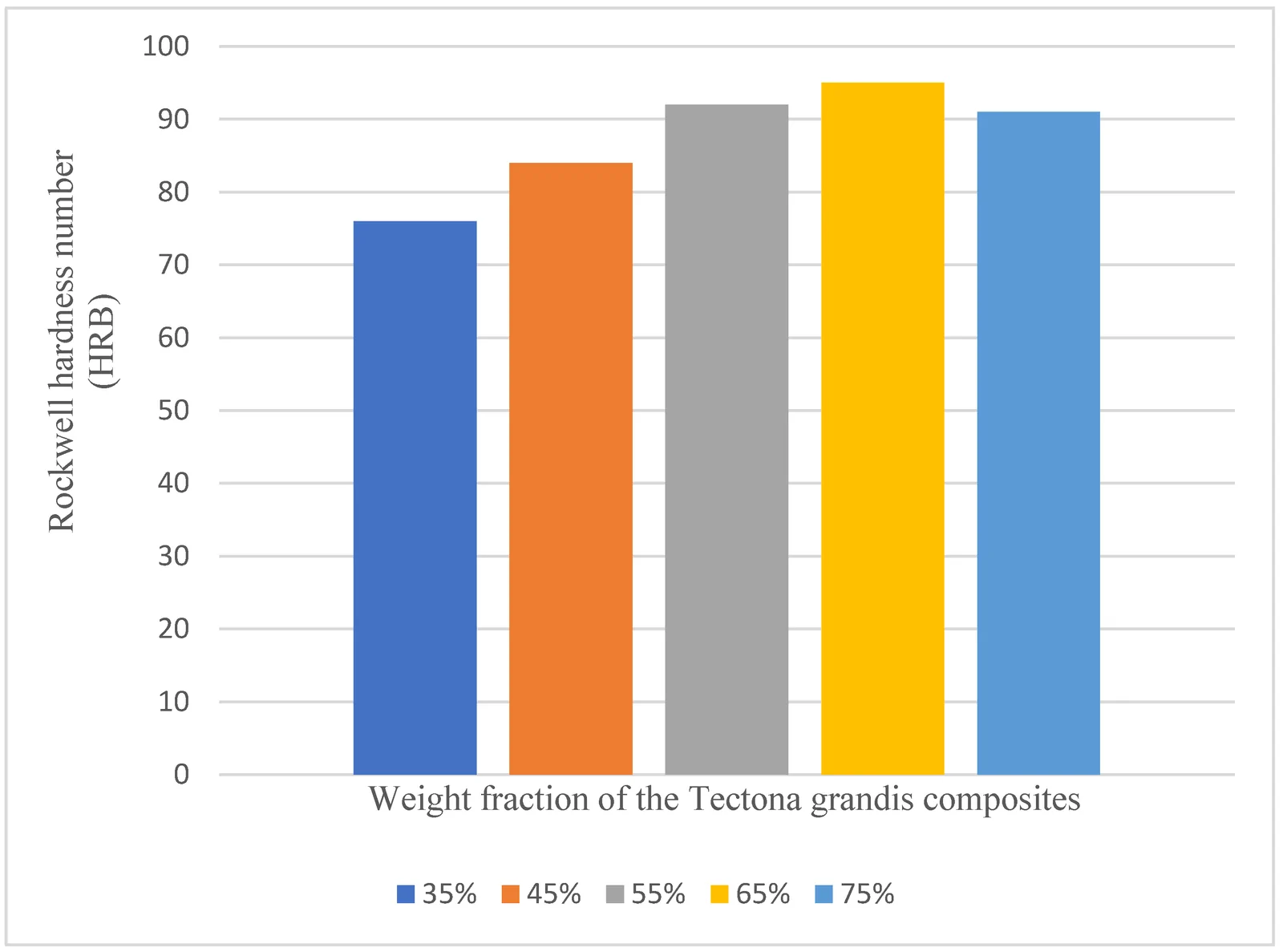
Correlation between Rockwell hardness number and weight fraction of Tectona grandis wood powder fiber composites.

The hardness number of Tectona grandis wood fiber composites, as observed in the experimental results shown in Figure 6, varies according to different fiber weight fractions. A noticeable trend emerges where the hardness decreases as the weight fraction of the fiber is reduced, highlighting the importance of fiber content in enhancing the composite’s surface hardness. This observation underscores the need to optimize fiber concentration to achieve the desired hardness levels for specific applications [36]. Initially, the hardness value was recorded at 76 HRB, but, with a 10% increase in fiber content, the hardness increased to 84 HRB. The increase in hardness with fiber content is due to the inherently rigid nature of the fibers, which reinforces the composite material. At higher filler content, the surface becomes more fiber-dominated, contributing to the increase in hardness. Table 4 presents the detailed hardness values for various weight fractions tested in this study.

### 3.4. Effect of Composite Fiber Reinforcement on Impact Strength

The Charpy impact test is the most widely adopted technique for assessing the impact strength of composite materials [37]. This test evaluates how well the material can absorb energy during a high-velocity impact, offering important understanding of its toughness and ability to withstand sudden forces. This test involves applying a sudden shock load to the composite specimens and measuring the energy absorbed during fracture. Appropriate reinforcement and processing allow composites to have even higher impact strength than components of the base matrix, because reinforcements act as barriers to crack formation and growth. This enhanced resistance is crucial for applications like sports equipment, automotive parts, and structures exposed to dynamic forces. The experimental findings show that, as the weight fraction increases, the impact strength improves accordingly. Initially, the impact strength was 236 J, while, with the 10% increase in the weight fraction, the obtained impact strength was 251 J. The increase in impact strength at intermediate fiber loadings is due to the energy absorption capacity of the fibers under impact. However, at very high fiber fractions, the impact strength stabilizes, and the reduced matrix content limits the energy dissipation mechanism. The impact strength of all the tested composites is presented in Table 5. This illustrates how changes in the wood fiber weight percentage influence the overall impact strength of the composite material. This analysis is crucial in establishing a good correlation between fiber content and mechanical characterization of the composite in order to determine the best composition of the material.

### 3.5. Effect of Composite Fiber Reinforcement on Moisture Absorption

**Figure 7:**
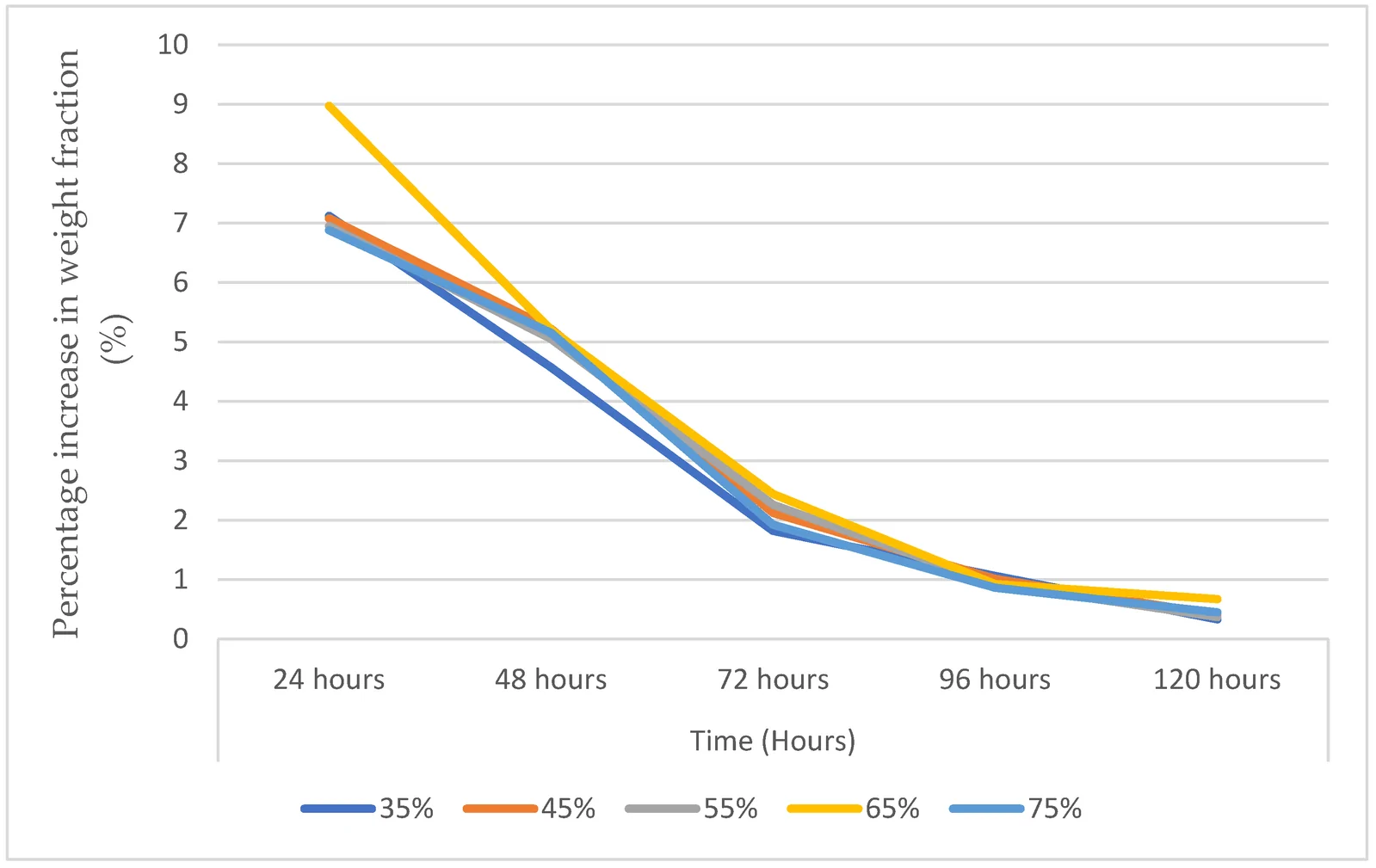
The percentage increase in weight fraction for Tectona grandis wood powder fiber composites.

The water absorption pattern of Tectona grandis wood fiber composites, as influenced by different weight fractions, is detailed in Figure 7. The influence of different fiber concentrations on the composite’s ability to absorb moisture is highlighted in this figure. There arose an observation that, the more the liquids were soaked in the solution, the more they absorbed moisture. Specifically, the composites with a 35% filler weight fraction exhibited an increased water absorption capability compared to those with lower filler weight fractions at the same immersion duration. It was observed that the moisture content of the materials tended to decrease with an increase in the fiber weight fraction; however, this trend became more pronounced only at higher immersion durations. The interaction between the fiber content and the matrix, where higher fiber content might initially result in increased voids or water pathways, could eventually reduce overall water absorption due to the hydrophobic nature of the matrix [38]. This is the case because Tectona grandis wood fibers are highly hydrophilic and possess open free hydroxyl groups in the cellulose and lignin components. These groups share forces with water molecules known as hydrogen bonds, thus causing moisture to be locked within the cell wall of the fiber. In Table 6, different samples’ initial weights and their weight after some specified intervals of time are mentioned. The changes in moisture content of the composites are taken as percentage changes in weight and can be determined from Equation (1), where the initial and final weights of the material are compared over different time intervals. This calculation method gives the quantitative determination of the absorption of water by the composite, thus giving users valuable information on the behavior of the composite when exposed to moisture for longer durations.

(1) $$=\frac{\left(Final\;Weight-Intial\;Weight\right)}{Intial\;weight}\times 100$$

For instance, a sample of Tectona grandis wood fiber composite with a 35% weight fraction initially weighed 15.72 g and increased to 18.18 g after 120 h of exposure. The progression of weight percentage changes over different time intervals is detailed in Table 6, demonstrating the moisture absorption behavior of the prepared composite materials. The moisture content, expressed as a percentage, can be calculated using the initial and final weights of the sample.

$$\begin{array}{l} =\frac{\left(16.84-15.72\right)}{15.72}\times 100\\ =7.12\% \end{array}$$

When the test begins, it is possible to observe high values of water absorption rate, and this indicates that the material at the start time can absorb as much water as possible due to the flow of the pores. The absorption level of the composite may take several hours or even days to reach its peak and eventually stabilize, demonstrating that the material has reached its saturation point, and any further water uptake will be minimal. This saturation period is influenced by the type of material and its degree of porosity. Untreated natural fibers, which are highly hydrophilic, are particularly prone to absorbing moisture. This characteristic leads to fiber swelling, which can compromise the integrity of the composite material by weakening the bond strength between the matrix and the fibers. This rise in moisture uptake is detrimental to the composite’s mechanical properties, particularly its tensile strength and hardness, especially at a higher fiber content. Applying appropriate chemical treatments to fibers and selecting suitable matrices can reduce these negative effects. Table 7 presents the percentage increase in weight, illustrating the variations in weight gain across different time periods.

### 3.6. Surface Morphology of Composites

**Figure 8:**
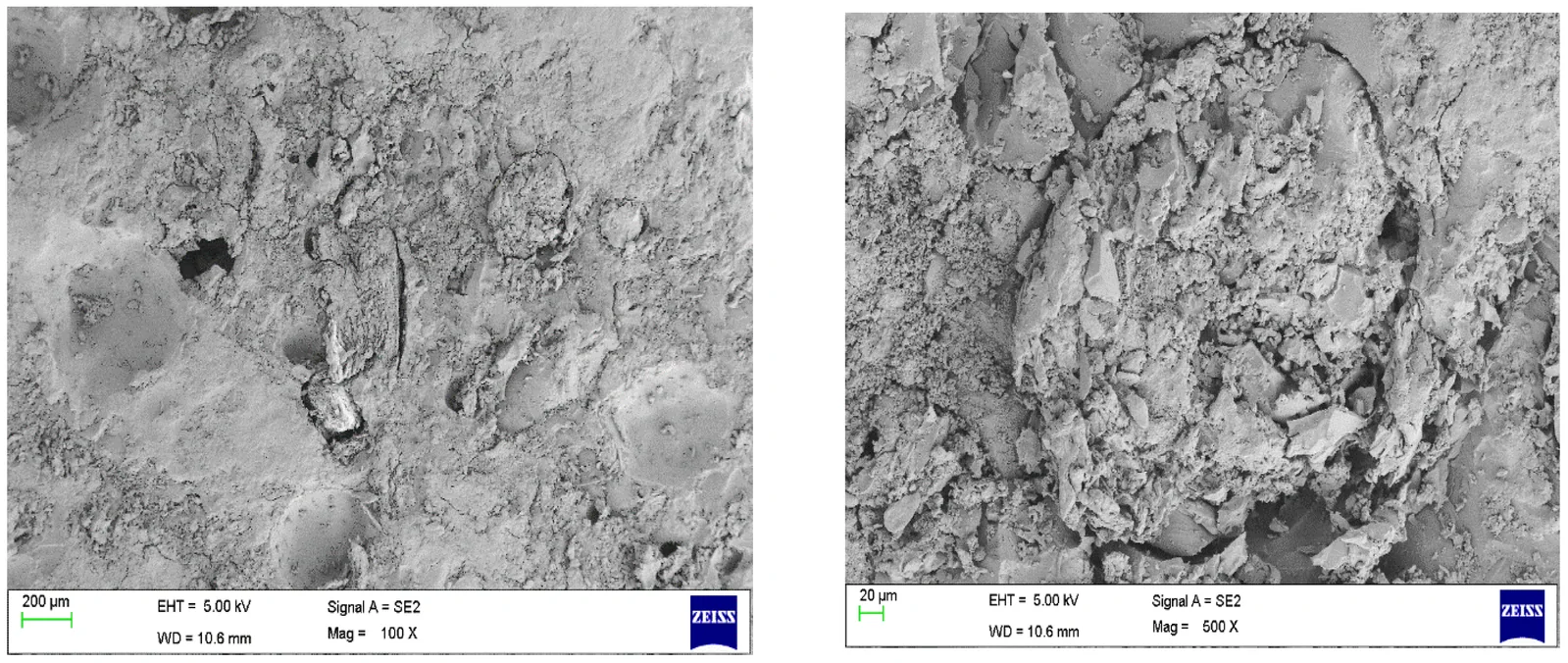
SEM images of the fracture surface of Tectona grandis wood powder composites, highlighting their structural features.

The SEM analysis of the tensile fractured surfaces revealed improved adhesion at the fiber–matrix interface, as indicated by fewer pull-outs and better fiber embedding in the matrix. This suggests a stronger bond between the fibers and the matrix, contributing to the enhanced mechanical properties observed in the composites. SEM images of the progressively damaged surfaces helped in identifying how material removal occurs during failure [39]. For Tectona grandis wood composites, as seen in Figure 8, the fracture surface reveals minimal wood powder pull-out. This observation indicates a relatively strong fiber–matrix interface adhesion, as fewer pull-outs generally signify better bonding between the fiber and the matrix.

**Figure 9:**
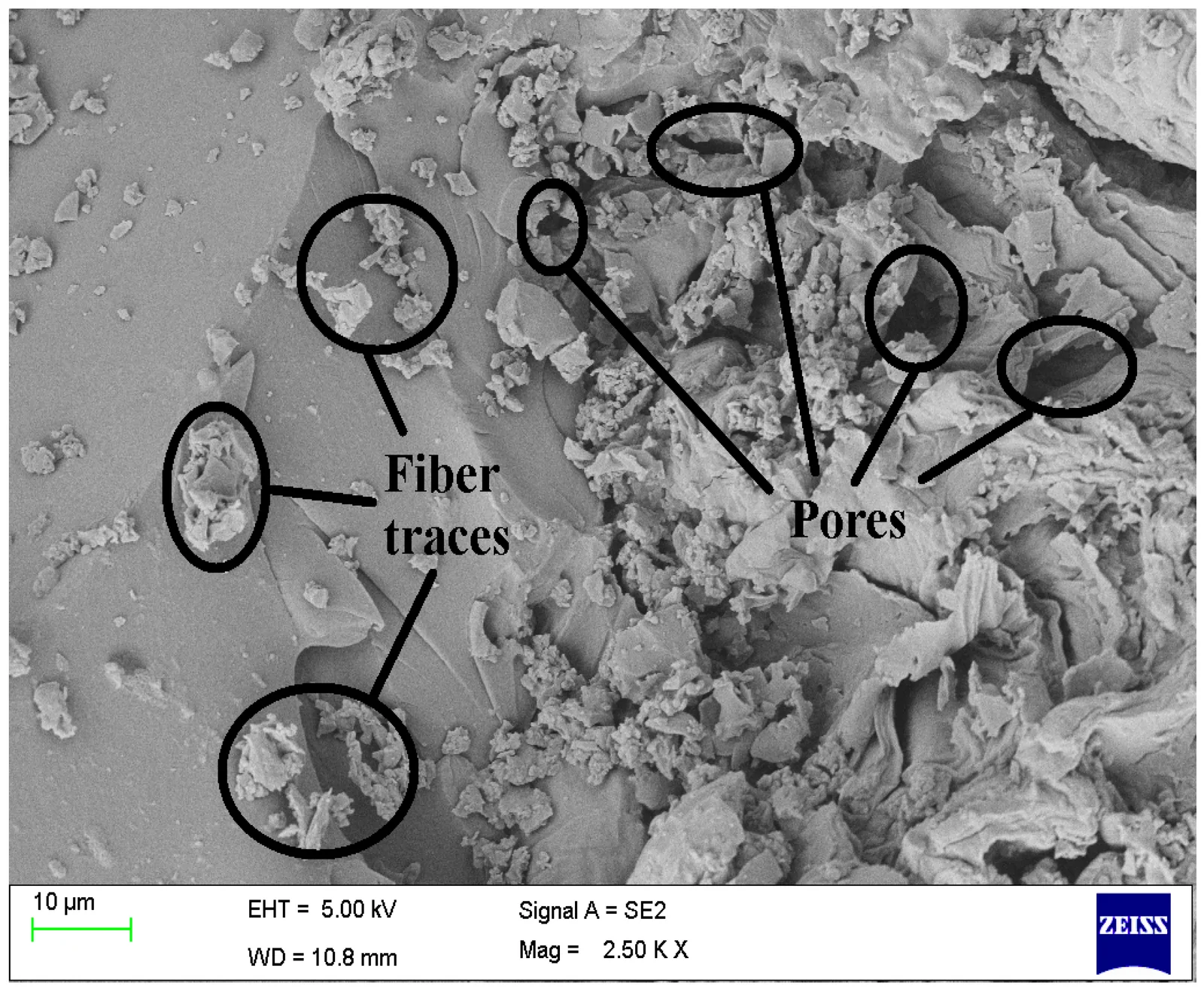
SEM image depicting the pores and traces of fiber in Tectona grandis wood powder composites.

The fiber surfaces appear smooth and crimped, with solid textures visible under magnification. This special morphology, commonly observed in natural fibers, suggests that Tectona grandis wood powder fiber composites could be useful, although weak fiber–matrix adhesion may limit their performance. Figure 9 also reveals more considerable pores, signifying that, although the integration of Tectona grandis wood powder raises the stiffness of the manufactured composite, the bonding interaction between particles and matrix is still weak.

The observed smooth fiber surfaces in the SEM images indicate poor fiber–matrix adhesion. This lack of adhesion limits the effective transfer of stress from the matrix to the fibers, which could contribute to the observed decline in tensile strength at higher fiber loadings. Inadequate pressure during the molding process and incomplete resin infiltration during curing could leave voids in the composite. Pores act as stress concentrators, which can weaken the composite by reducing its load-bearing capacity.

### 3.7. Static Analysis by ANSYS

**Figure 10:**
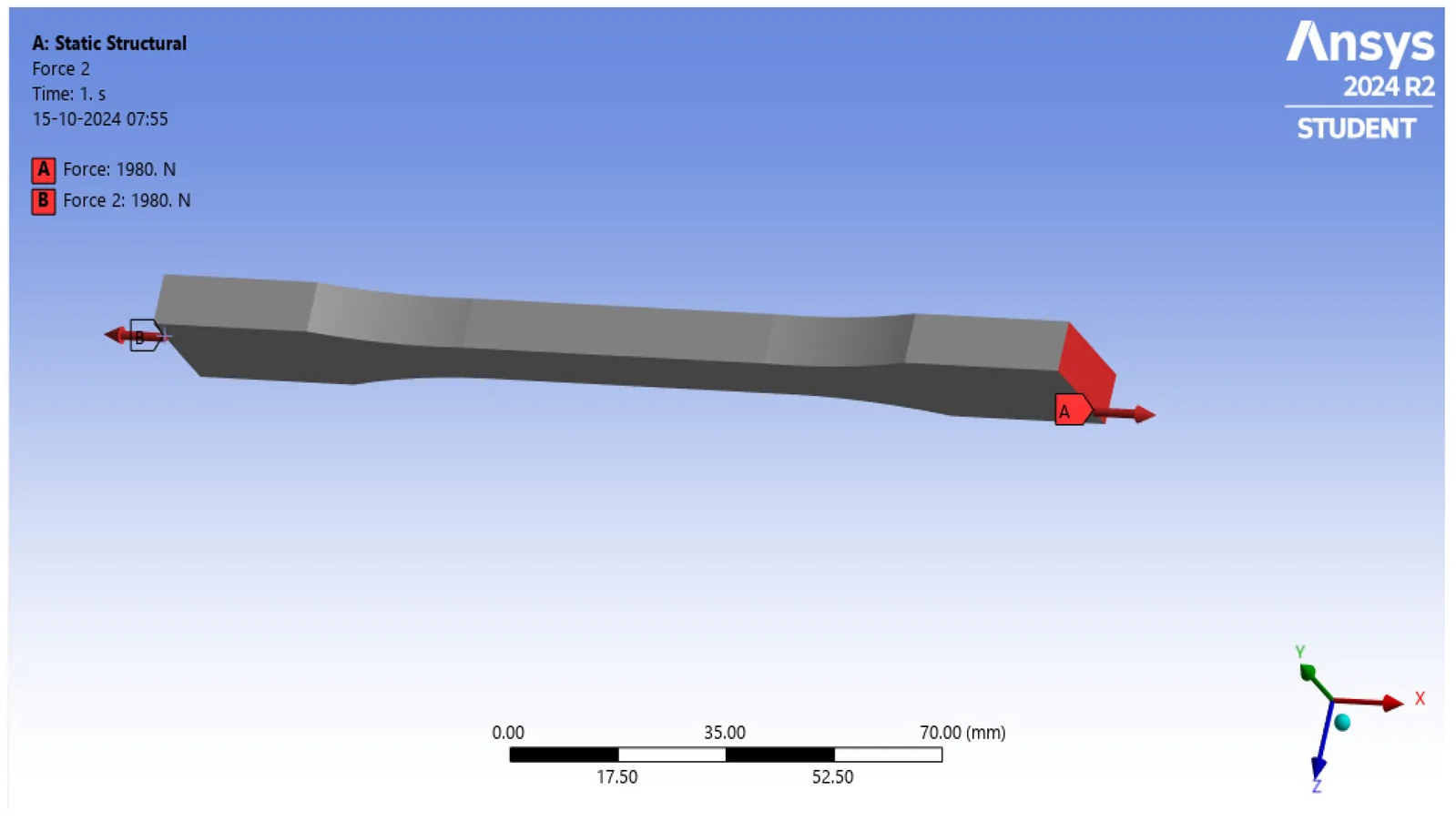
Illustration of the application of a uniaxial tensile load at both ends of the specimen.

**Figure 11:**
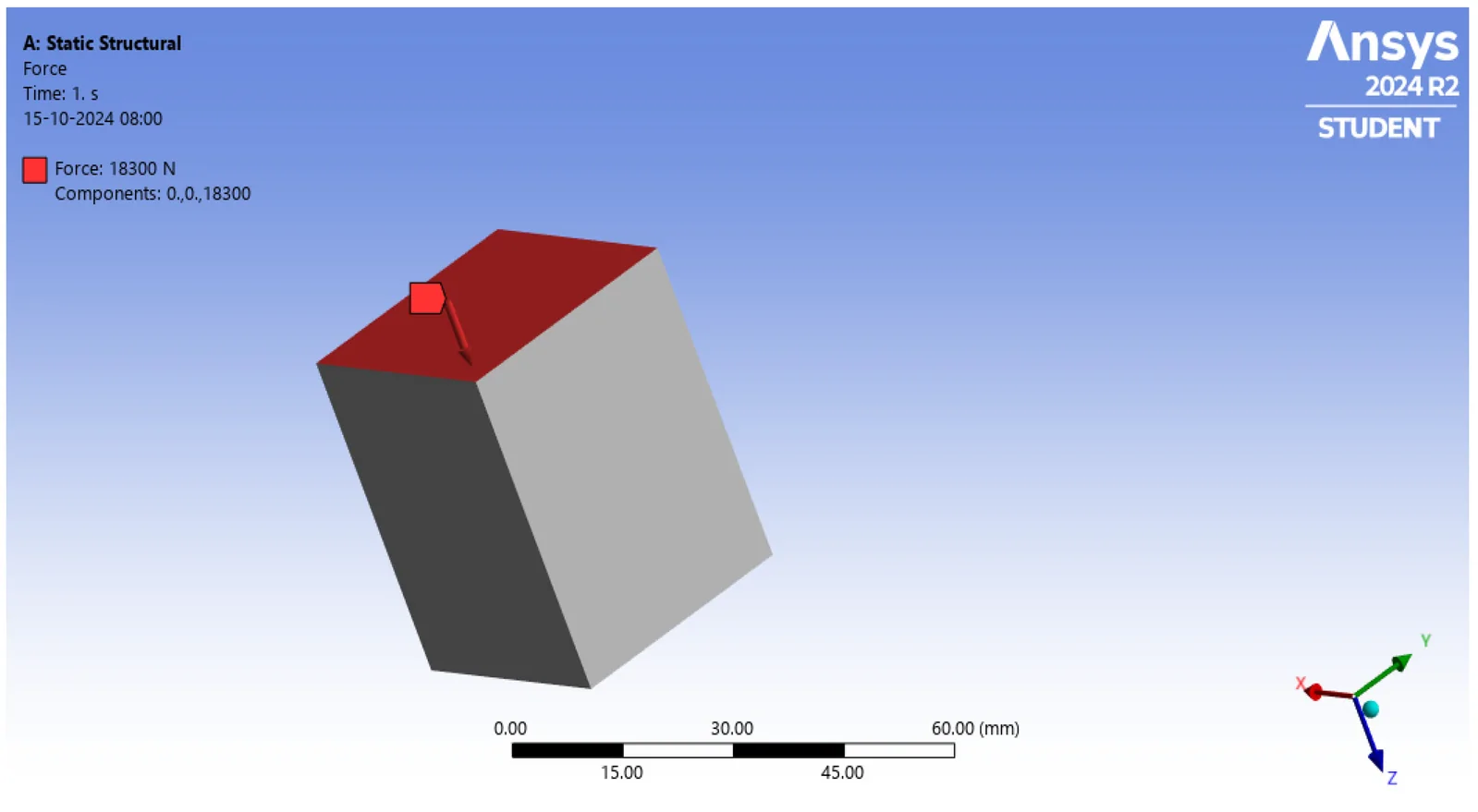
Uniaxial compression load applied at one end while the opposite end is fixed.

The initial step in ANSYS involves creating a precise model, followed by generating a refined mesh. A tetrahedral element is used to capture the stress distribution accurately for the composite structure. The boundary conditions used in the FEA simulations involved zero displacements in all directions and applying a uniform tensile load at both ends. The simulations conducted in ANSYS revealed critical information about stress distribution and deformation behavior under varying load scenarios. The areas where high-stress concentrations occur coincide with the points of failure noted in physical–mechanical tests. These results provided valuable insights into the load transfer mechanisms within the composite and help pinpoint the ideal fiber content for optimal performance. Material properties, such as elastic modulus and Poisson’s ratio, were derived from the experimental results. For composites with varying fiber fractions, the respective experimental values were assigned in the simulation. In the case of tensile and compression specimens, the load was applied along the Z-axis to determine the mechanical characteristics, as seen in Figure 10 and Figure 11. The tensile test requires creating force on the specimen shaped like a dumbbell to decide tensile strength and to check if it could endure elongation, while the compression test puts the load on the cubic specimen to determine the strength and the extent to which it could elastically deform.

Following establishment of the boundary conditions, to ensure accurate analysis, the composite models were solved with a high level of precision. Subsequently, the total deformation and von Mises stress of the composites were calculated to evaluate their mechanical response under applied loads [40]. For Tectona grandis wood powder composites with a 35% weight fraction, the von Mises stress was found to be 7.732 MPa. Detailed data on the total deformation and equivalent stress of both tensile and compression specimens, along with the weight fractions of the various composites analyzed in this study, are comprehensively presented in Table 8 and Table 9. These tables provide an in-depth overview of the mechanical response of the composites under different loading conditions, illustrating how variations in fiber weight fractions influence their stress distribution and deformation behavior. The simulation results showed a good match with the experimental results for composites with lower and moderate fiber fractions. However, discrepancies at higher fiber fractions are due to issues such as fiber agglomeration and poor fiber–matrix adhesion.

**Figure 12:**
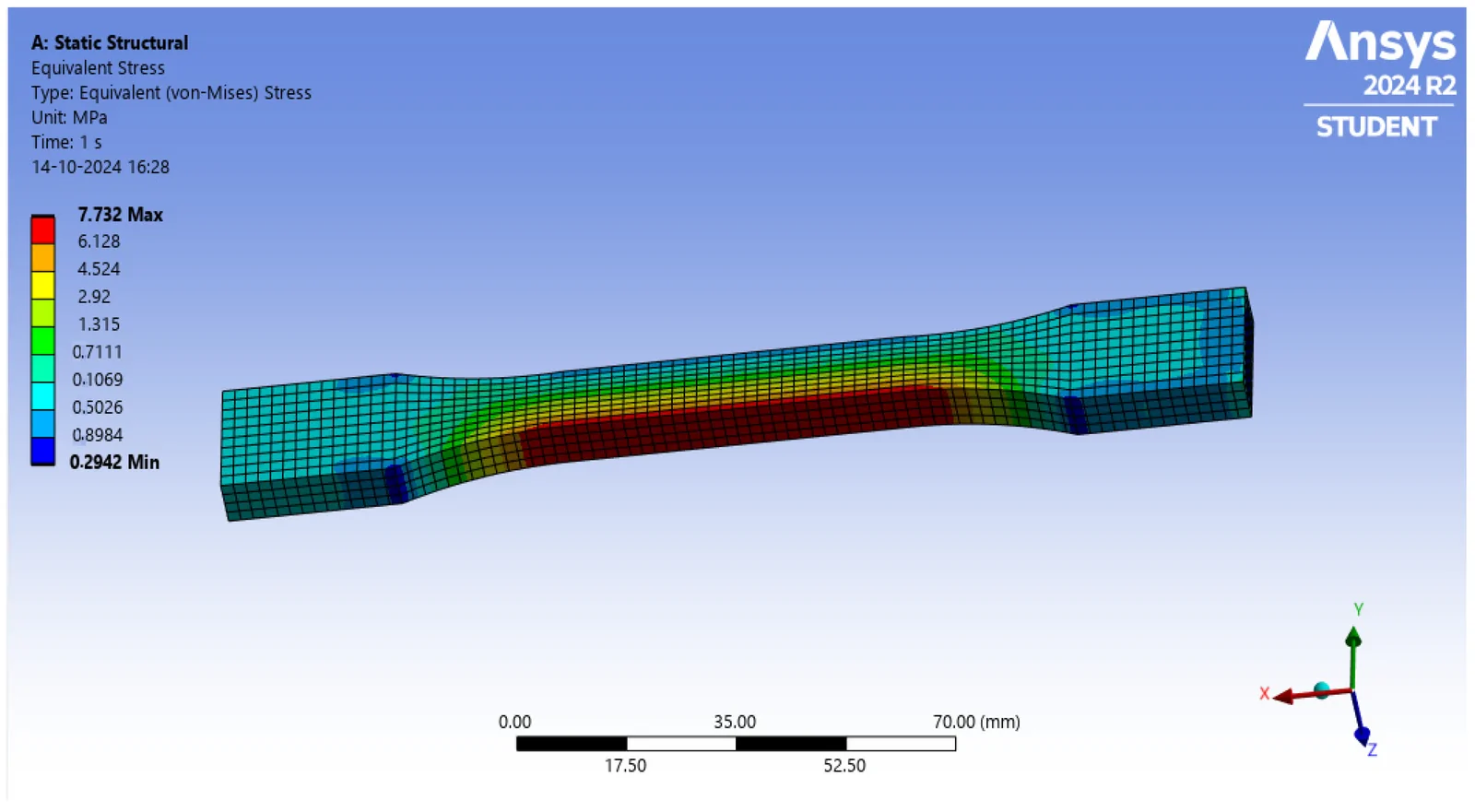
Distribution of equivalent stress in Tectona grandis wood powder composites as observed in the tensile specimen.

**Figure 13:**
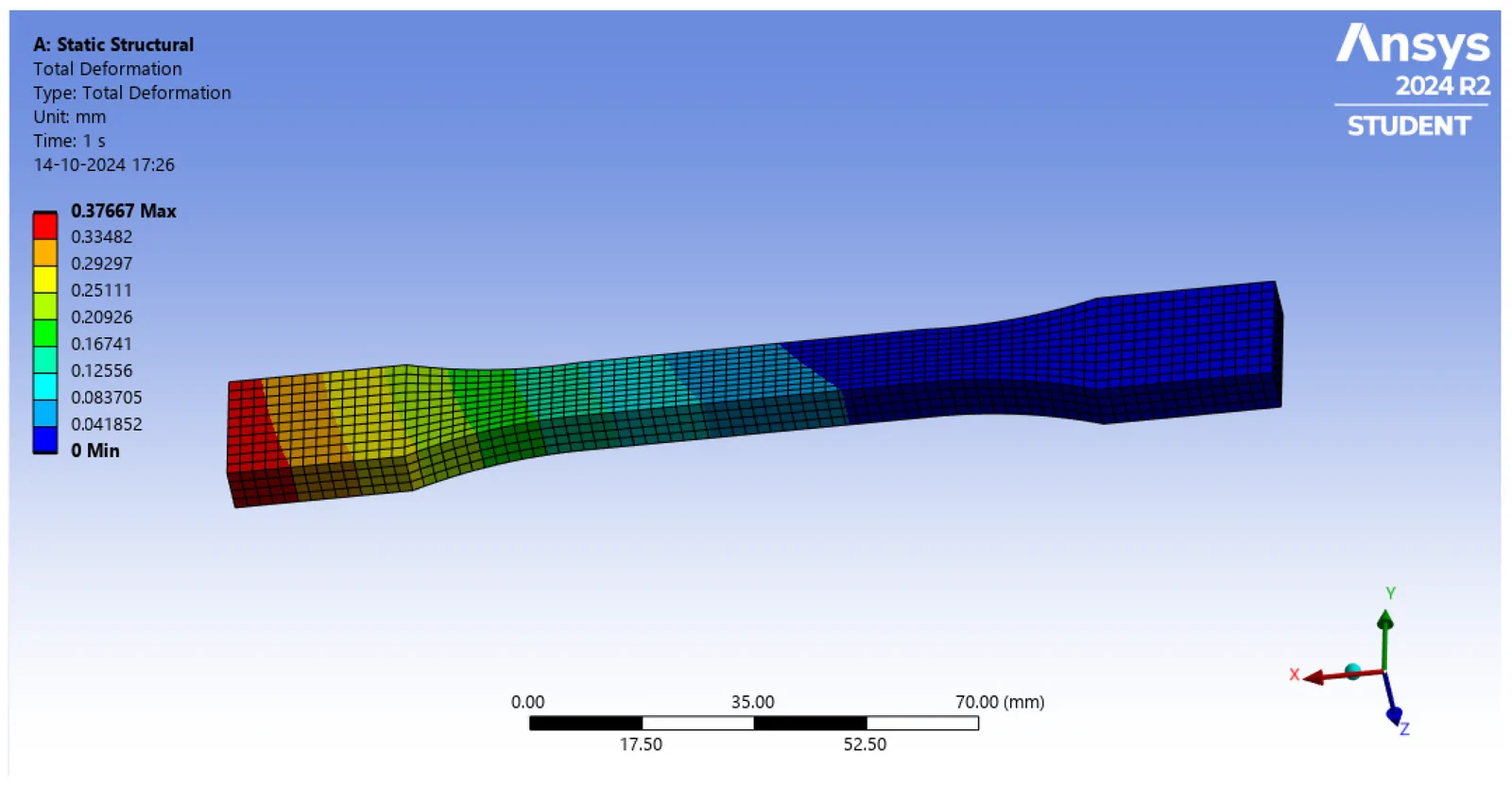
Total deformation observed in Tectona grandis wood powder composites within the tensile specimen.

Figure 12 and Figure 13 depict the equivalent (von Mises) stress and total deformation of Tectona grandis composites containing 35 wt % material. When complex loading conditions are applied to isotropic and brittle materials, the equivalent stress criterion is commonly employed to determine the yielding property of the material. As shown for the tensile strength, the maximum stress concentration was observed in the web region of the specimen, indicating the area most susceptible to failure under the applied load [41]. Because of these fluctuating geometries, the web area becomes smaller, and hence, a failure occurs in that web area. The distribution of stress in the web region of the specimen was notably higher than in other areas. As a result, the stress levels in the rest of the specimen were significantly lower. If a uniaxial tensile load is applied from two ends, the web region’s stress decreases, and when the material reaches the yield point, it becomes brittle, thereby showing signs of failure.

When placed under an external load, the specimens deform, and the total deformation is maximal in the neighborhood of the ends. For Tectona grandis wood powder composites, the maximum deformation reaches 0.376 mm at the ends, while the minimum deformation occurs at the center, measuring 0.00418 mm. As the applied load increases, the deformation also increases, which is attributed to the 10% increase in weight fraction. This change in weight fraction requires a higher load for the material to reach failure, enhancing the composite’s load-bearing capacity.

**Figure 14:**
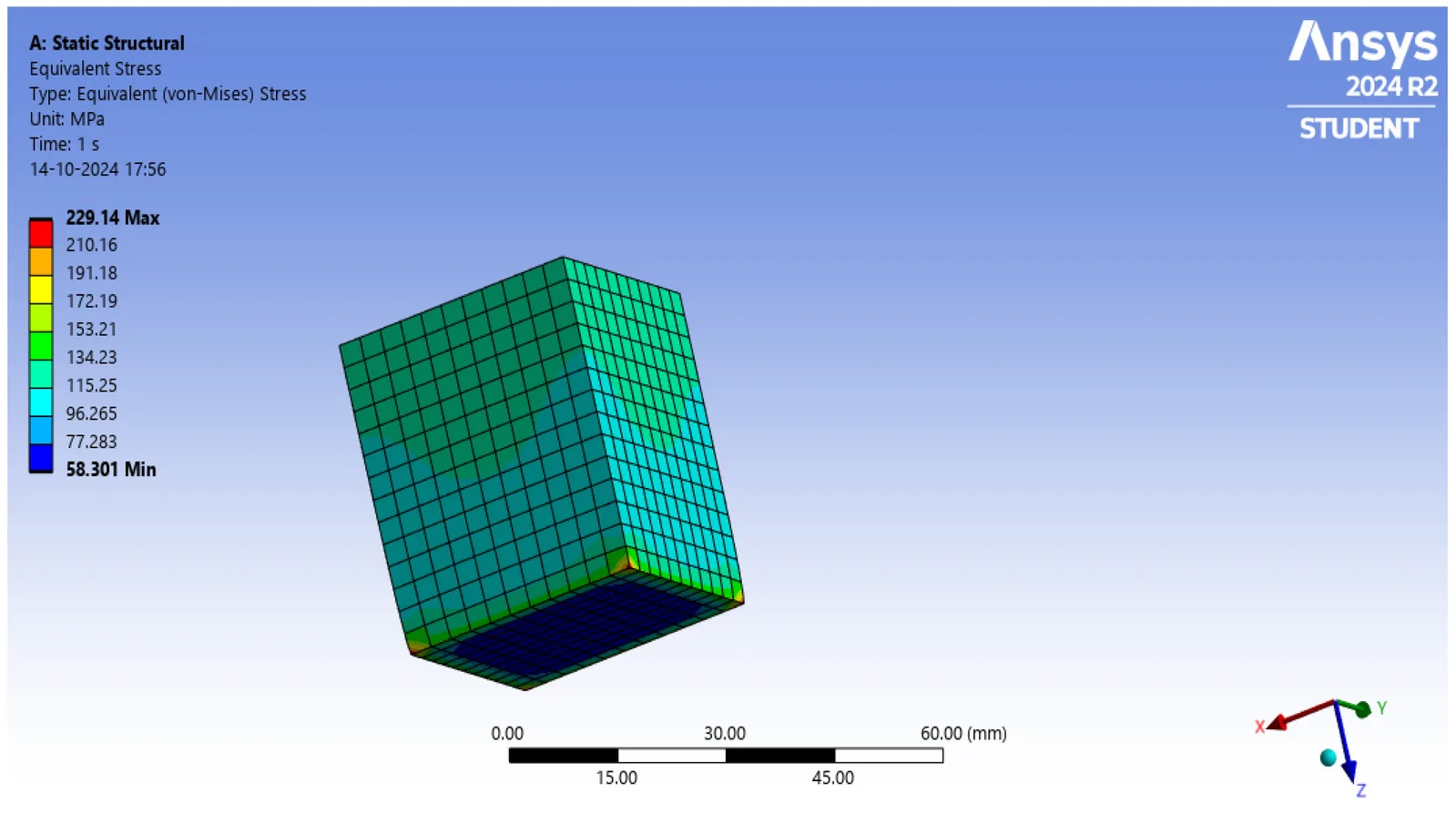
Distribution of equivalent stress in Tectona grandis wood powder composites observed in th compression specimen.

**Figure 15:**
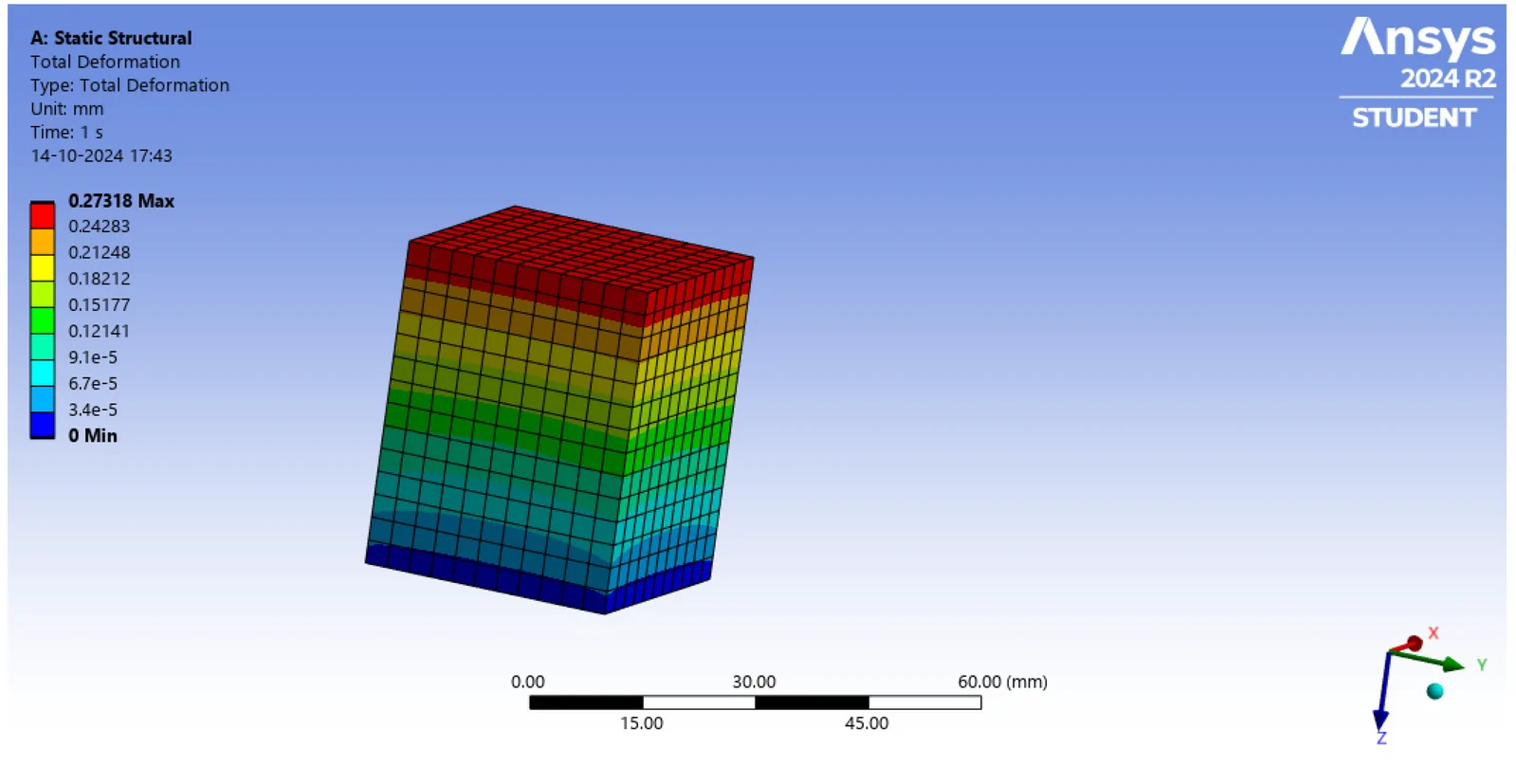
Total deformation exhibited by Tectona grandis wood powder composites in the compression specimen.

Figure 14 and Figure 15 display the equivalent (von Mises) stress and total deformation of the compression specimens of Tectona grandis wood powder composites that contained a 35% weight fraction. One end of the specimen is restrained from any movement, whilst a compressive force is applied in the axial direction of the surface. The equivalent stress of 229.14 MPa occurs on the side opposite the load application. Table 9 summarizes the compression specimens and weight fractions by their equivalent stress and deformation. The failure of the specimen can be attributed to the uniform load distribution across the surface, and sharp corners cause the material to fail at those points. The stress concentration is highest at one corner, as highlighted in red. The overall deformation, as depicted in another figure, rises to the top surface of the load application with a maximum of 0.273 mm.

## 4. Conclusions

The fabrication and characterization of composite materials reinforced with Tectona grandis wood powder fibers have yielded significant insights into their mechanical performance and potential applications. Using a compression molding technique, the fibers were successfully integrated into a polymer matrix, resulting in composite samples with enhanced mechanical properties. These samples were tested for tensile strength, compressive strength, hardness, impact resistance, and moisture absorption, following ASTM standards to ensure reliability and accuracy. The results demonstrated that incorporating Tectona grandis wood powder fibers improved the overall mechanical characteristics of the composites. This improvement underscores the viability of using natural fibers as reinforcements due to their eco-friendly, renewable, and biodegradable nature. Natural fibers not only offer environmental benefits but also contribute to the production of lightweight composites with improved specific mechanical properties. This makes them suitable for diverse applications across industries, such as the aerospace, automotive, and construction industries.

Therefore, the following conclusions can be drawn from the results obtained from the experiments:

- The fiber used in the composite formulation, Tectona grandis wood powder fiber, increased the overall mechanical properties of the composite with a tensile strength of 18.68 MPa and a compressive strength of 249.83 MPa. At the same time, much better impact resistance was achieved due to the capacity of fibers to absorb energy.
- Analysis of the SEM results provided a consistent fiber–matrix interface when the fiber content was at an optimum level: a condition that enhanced the mechanical behavior of the composite system.
- The moisture absorption test showed minimal weight change in the Tectona grandis wood powder composite after 120 h of water immersion, indicating the fibers’ hydrophilic nature. This highlights the need for fiber treatment and matrix optimization to reduce moisture retention.

## Figures and Tables

**Table 1:** Variable characteristics of the composite panels.

Composite	The Weight Fractions of the Prepared Composite Materials
Composite 1	35% of Tectona grandis wood powder fibers + 65% of epoxy resin
Composite 2	45% of Tectona grandis wood powder fibers + 55% of epoxy resin
Composite 3	55% of Tectona grandis wood powder fibers + 45% of epoxy resin
Composite 4	65% of Tectona grandis wood powder fibers + 35% of epoxy resin
Composite 5	75% of Tectona grandis wood powder fibers + 25% of epoxy resin

**Table 2:** Findings from the tensile test.

Composites	Weight Fraction (%)	Ultimate Load (N)	Standard Deviation (N)	Tensile Strength (MPa)	Strain at Break (%)	Young’s Modulus (GPa)
Tectona grandis wood powder fiber	35	1980	260	8.11	2.3	5.8
	45	2430	95	8.49	2.1	6.9
	55	3970	111	12.19	1.8	7.4
	65	4680	86	18.68	1.6	8.5
	75	4420	127	17.13	1.3	7.9

**Table 3:** Summary of the results obtained from the compression test.

Composites	Weight Fraction (%)	Ultimate Load (N)	Standard Deviation (N)	Compression Strength (MPa)	Strain at Break (%)	Young’s Modulus (GPa)
Tectona grandis wood powder fiber	35	18,300	984	196.32	5.3	5.6
	45	20,900	1113	209.50	4.9	6.8
	55	23,200	754	225.98	4.7	7.3
	65	24,900	360	249.83	4.3	8.2
	75	24,600	1081	243.17	4.1	7.6

**Table 4:** Hardness numbers of Tectona grandis fiber composites.

Composites	Weight Fraction (%)	Load (Kg)	Rockwell Hardness Number (HRB)
Tectona grandis wood powder fiber	35	60	76
	45	60	84
	55	60	92
	65	60	95
	75	60	91

**Table 5:** Impact strength values for Tectona grandis wood powder fiber composites.

Composites	Weight Fraction (%)	Impact Strength (J)
Tectona grandis wood powder fiber	35	236
	45	251
	55	268
	65	262
	75	257

**Table 6:** Weight of the moisture-absorbed composites.

Composites	Weight Fraction (%)	Initial Weight (g)	Weight after 24 h	Weight after 48 h	Weight after 72 h	Weight after 96 h	Weight after 120 h
Tectona grandis wood powder fiber	35	15.72	16.84	17.61	17.93	18.12	18.18
	45	14.68	15.72	16.54	16.89	17.06	17.13
	55	16.12	17.24	18.11	18.52	18.69	18.76
	65	13.93	15.18	15.97	16.36	16.51	16.62
	75	15.26	16.31	17.15	17.48	17.63	17.71

**Table 7:** The percentage of weight increase over the specified period.

Composites	Weight Fraction (%)	Percentage Increase in Weight				
		24 h	48 h	72 h	96 h	120 h
Tectona grandis wood powder fiber	35	7.12	4.57	1.82	1.06	0.33
	45	7.08	5.22	2.12	1.01	0.41
	55	6.95	5.05	2.26	0.92	0.37
	65	8.97	5.20	2.44	0.92	0.67
	75	6.88	5.15	1.92	0.86	0.45

**Table 8:** Analytical results obtained from the tensile specimens.

Weight Fraction	Ultimate Load (N)	Total Equivalent Stress (MPa)	Deformation (mm)
35%	1980	7.732	0.376
45%	2430	8.24	0.423
55%	3970	10.31	0.561
65%	4680	17.98	0.687
75%	4420	16.76	0.526

**Table 9:** Analytical results obtained from the compression specimens.

Weight Fraction	Ultimate Load (N)	Total Equivalent Stress (MPa)	Deformation (mm)
35%	18,300	229.14	0.273
45%	20,900	237.13	0.293
55%	23,200	248.43	0.346
65%	24,900	261.78	0.391
75%	24,600	255.22	0.364

## Data Availability

The data will be made immediately available on request.
