# Correction: Bavanam Nagaraja Reddy et al. Development and Evaluation of a Polymer Composite Material Reinforced by Tectona Grandis Fiber, with Static Analysis. *Polymers* 2025, *17*, 634

**DOI:** 10.3390/polym18141715

**Published:** 2026-07-13

**Authors:** Sandeep Bavanam Nagaraja Reddy, Kishor Buddha, Kadiyala Chandra Babu Naidu, Dudekula Baba Basha

**Affiliations:** 1 Department of Mechanical Engineering, School of Technology, GITAM University, Bangalore 562163, Karnataka, India; snagaraj2@gitam.edu; 2 Department of Physics, GITAM Deemed to be University, Bangalore 561203, Karnataka, India; chandrababu954@gmail.com; 3 Department of Information Systems, College of Computer and Information Sciences, Majmaah University, Al’Majmaah 11952, Saudi Arabia

There was an error in the original publication [1]. There was a sentence in paragraph 7 from the Section 1 Introduction in the original publication that read

“Accordingly, this study utilized weight fractions of 35%, 45%, 55%, 65%, and 75% for the composites.”

A correction has been made to this sentence, which currently reads

“Accordingly, the present study builds upon the preliminary results published in [15], including additional data and a more comprehensive analysis, where weight fractions of 35%, 45%, 55%, 65%, and 75% were utilized for the composites.”

The authors state that the scientific conclusions are unaffected. This correction was approved by the Academic Editor. The original publication has also been updated.
